# Editorial: Artificial Intelligence for Medical Image Analysis of Neuroimaging Data

**DOI:** 10.3389/fnins.2020.00480

**Published:** 2020-05-21

**Authors:** Nianyin Zeng, Siyang Zuo, Guoyan Zheng, Yangming Ou, Tong Tong

**Affiliations:** ^1^Department of Instrumental and Electrical Engineering, Xiamen University, Xiamen, China; ^2^Key Laboratory of Mechanism Theory and Equipment Design, Ministry of Education, Tianjin University, Tianjin, China; ^3^School of Biomedical Engineering, Institute of Medical Robotics, Shanghai Jiao Tong University, Shanghai, China; ^4^Department of Radiology, and Computational Health Informatics Program, Boston Children's Hospital, Boston, MA, United States; ^5^College of Physics and Information Engineering, Fuzhou University, Fuzhou, China

**Keywords:** medical image analysis, artificial intelligence, machine learning, pattern recognition, computational intelligence

With the development of advanced medical imaging techniques, a huge amount of medical images have been produced in various healthcare institutes and hospitals. Especially, there is a growing research interest in a more multidisciplinary approach for investigating brain structure and function in living humans and animals. In order to better interpret brain images, there is an increasing demand to introduce artificial intelligence methods such as machine learning, expert systems, robotics and perception, and evolutionary computation to automatically exploit useful information besides visual features. It should be pointed out that brain images themselves exhibit several distinguishing features that add to the difficulties in their analysis. In recent years, there have been many new research achievements in each aspect of artificial intelligence for brain image analysis. This Research Topic sought original contributions that address the challenges of artificial intelligence for brain image analysis and welcomed researchers in this field to share their experiences and new research achievements.

We were pleased to receive many submissions from authors of their latest research results on artificial intelligence methods for medical image analysis. Nineteen papers are finally accepted from a total of 29 submissions after rigorous reviews. They were contributed from different countries and regions, including China, the United Kingdom, the United States, Germany, South Korea, Denmark, Canada, and more.

Here, a brief introduction of the 19 accepted papers is given. We refer the readers to the papers in this topic and the references therein for more details. Lin W. et al. established a deep learning approach based on convolutional neural networks (CNN) to accurately predict MCI-to-AD conversion with magnetic resonance imaging (MRI) data. Kazeminejad and Sotero introduced a new biomarker extraction pipeline for Autism Spectrum Disorder that relies on the use of graph-theoretical metrics of fMRI-based functional connectivity to inform a support vector machine. Bi et al. proposed an advanced method, namely an evolutionary weighted random support vector machine cluster, for analysis of Alzheimer's disease. Ladefoged et al. focused on the problem of attenuation correction of PET/MRI in pediatric brain tumor patients based on a deep learning method. Livne et al. established a U-Net deep learning framework for high-performance vessel segmentation in patients with cerebrovascular disease. Wang, Sun et al. proposed a 14-layer convolutional neural network for the identification of multiple sclerosis. Huang C. et al. developed a new fusion method based on the combination of the shuffled frog leaping algorithm and a pulse coupled neural network for the fusion of SPECT images and CT images to improve the quality of fused brain images. Xin et al. utilized a deep learning method to find differences between the brains of men and women. Zhang Y. et al. proposed an improved wavelet threshold for image de-noising. Lin C. et al. proposed a novel low-rank method for the simultaneous recovery and segmentation of pathological MR brain images. Zhang Z. et al. developed a multi-scale time-series model for the diagnosis of brain diseases. Gupta et al. proposed a novel machine learning-based framework to discriminate subjects with AD or MCI, utilizing a combination of four different biomarkers. Zhao et al. proposed a supervised brain tumor segmentation method based on gradient and context-sensitive features. Huang Y. et al. developed a multi-modality 3D convolutional neural network for the diagnosis of Alzheimer's disease. Wang L. et al. presented the use of Nested Dilation Networks for brain tumor segmentation. Gwo et al. developed a method to characterize and quantify the shape, texture, and potential growth of white matter hyperintensity lesions. Xu et al. introduced a fully automatic framework for Parkinson's disease diagnosis. Wang, Xie et al. proposed an AlexNet transfer learning model for alcoholism identification. Wang, Tang et al. developed a densely connected neural network for analysis of cerebral micro-bleeding.

In the end, we strongly hope that this Research Topic will attract more research attention to artificial intelligence methods for medical image analysis. We thank the reviewers for their efforts to guarantee the high quality of this collection. We also thank all of the authors who have contributed.

## Author Contributions

NZ wrote the editorial. SZ, GZ, YO, and TT edited the editorial.

### Conflict of Interest

The authors declare that the research was conducted in the absence of any commercial or financial relationships that could be construed as a potential conflict of interest.

